# Optimizing Cardiovascular Treatment in Non-Small Cell Lung Cancer: A Comprehensive Computational Approach for Assessment of Drug-Drug Interactions Between Tyrosine Kinase Inhibitors and Cardiovascular Drugs

**DOI:** 10.12688/f1000research.162353.3

**Published:** 2026-02-02

**Authors:** Prajakta Patil, Mrunal Desai, Gayathri Baburaj, Levin Thomas, Viswam Subeesh, Sumit Birangal, Mahadev Rao, Gurupur Gautham Shenoy, Jagadish P. C.

**Affiliations:** 1 Department of Pharmaceutical Chemistry, Manipal College of Pharmaceutical Sciences, Manipal Academy of Higher Education, Manipal, Karnataka, 576104, India; 2 Department of Pharmacy Practice, Manipal College of Pharmaceutical Sciences, Manipal Academy of Higher Education, Manipal, Karnataka, 576104, India

**Keywords:** Cardiovascular drugs, Drug-drug interactions, NSCLC, IBM Micromedex, Drugs.com, Molecular docking, TKI

## Abstract

**Background:**

As lung cancer treatment has progressed, there has been an increase in awareness of the short- and long-term adverse effects of targeted cancer therapies of tyrosine kinase inhibitors, particularly cardiovascular toxicities.

**Methods:**

The current study assessed the potential drug-drug interactions using interaction checkers (IBM Micromedex and Drugs.com). Molecular docking was employed to further investigate the involvement of human ether-à-go-go-related gene (hERG) and pregnane X receptor (PXR) proteins to elucidate their potential interactions and their underlying mechanisms.

**Results:**

A total of 74 pharmacokinetic and 105 pharmacodynamic interactions were detected between tyrosine kinase inhibitors and cardiovascular drugs, along with a report on the severity and level of documentation. A considerable fraction of molecular modelling outcomes concurred with information from drug-drug interaction checkers. The binding energies of tyrosine kinase inhibitors with hERG and PXR were high, indicating significant interactions. The cardiovascular drug class encompasses calcium channel blockers, antiarrhythmic medicines, and statins, which were observed to exhibit synergistic interactions. The identification of these potential drug-drug interactions involving CYP3A4, P-gp, and hERG proteins can be utilized in therapy optimization in clinical settings.

**Conclusion:**

This study will aid clinicians in designing safe dosage regimens for patients with lung cancer. In cases where patients have multiple comorbidities, it is essential to study the clinical aspects to design efficient chemotherapy and manage adverse effects and toxicities.

## 1. Introduction

The global prevalence of lung cancer as the primary cause of cancer-induced mortality is expected to continue in the foreseeable future. According to the GLOBOCAN report 2020, lung cancer is the leading cause of new cancer cases worldwide, comprising 11.4% of all cancers and resulting in 18% of cancer-related fatalities. ^1^ Approximately 85% of lung tumors are non-small cells, and out of them, 60 to 70% of non-small cell lung cancer (NSCLC) patients exhibit stage III or IV disease. ^2^ The approval of multiple tyrosine kinase inhibitors (TKI) in the last decade has significantly transformed the management of NSCLC. More than 22 TKIs are currently being utilized in clinical settings or are undergoing advanced clinical trials to target oncogenic drivers of NSCLC, including EGFR, BRAF, MET, ALK fusion rearrangement, and ROS1-fusion rearrangement. ^3, 4^ Currently, several FDA-approved first-line therapies exist for patients with metastatic NSCLC with epidermal growth factor receptor (EGFR) exon 19 deletions or exon 21 L858R mutations, which work by competitively blocking ATP binding and subsequent phosphorylation of the EGFR tyrosine kinase domain. ^5– 7^

The objective of targeted therapies for NSCLC is to enhance anticancer efficacy while minimizing adverse effects compared to the traditional anticancer medications. This approach has yielded promising results, leading to a significant increase in the number of targeted therapeutic agents developed over the last decade. ^8^ Nevertheless, extensive utilization of this approach has raised concerns regarding its potential cardiotoxicity and off-target effects. Pathways that give rise to pathological survival and uncontrolled proliferation of cancer cells often affect the survival of normal cells, including cardiomyocytes. Inhibition of prosurvival kinases in regular cardiomyocytes may lead to cardiomyopathy, which is a clear manifestation of on-target cardiotoxicity when targeting these pathways in cancer cells. ^9^ The widespread use of TKIs has raised concerns regarding their potential cardiotoxicity and associated symptoms, including, heart failure, arrhythmia/QT prolongation, hypertension, and acute coronary syndrome/myocardial ischemia. Individuals with preexisting cardiac illnesses are at a heightened risk of cardiac damage. It has been observed that 23% of patients diagnosed with lung cancer also have underlying cardiovascular (CV) conditions. The risk of cardiovascular disease (CVD) is elevated in such individuals with lung cancer. The observed association could potentially be attributed to the presence of chronic inflammation in CVD. ^10, 11^

Recent pharmacoepidemiological investigations have increasingly highlighted the real-world cardiovascular safety concerns associated with TKIs, beyond what has been observed in controlled clinical trials. Most recently, a large pharmacovigilance analysis using the United States FDA Adverse Event Reporting System (FAERS) has provided robust real-world evidence on cardiovascular toxicities associated with selective ALK-specific TKIs as well as RET-specific TKIs, including pralsetinib and selpercatinib. This study identified significant proportionality for hypertension, ischemic heart disease, and torsade de pointes/QT prolongation, with median onset occurring within the first few weeks of treatment initiation. Notably, a substantial proportion of reported cases resulted in serious outcomes such as hospitalization and death, underscoring the clinical significance of these findings. Large-scale observational and post-marketing studies indicate that cardiovascular adverse events occur with notable frequency and often manifest early during therapy.

In response to this issue, various oncology and cardiology governing bodies in the United States and Europe have released guidelines regarding the monitoring of patients undergoing cancer treatments that may have cardiotoxic effects. These guidelines cover the management of CV toxicities and monitoring of CVD in patients with cancer. ^12^ Left ventricular dysfunction (LVD) and heart failure (HF) are prevalent markers of cardiotoxicity associated with anti-cancer therapies. The long-term use of crizotinib has been linked to bradycardia and QT prolongation. In a phase III clinical trial, it was observed that 69% of the patients experienced at least one episode of asymptomatic sinus bradycardia, which is characterized by a heart rate of less than 60 beats per minute. Additionally, 1.4% of patients exhibited QTc prolongation. ^13, 14^ Reports have indicated that TKIs that interfere with the vascular endothelial growth factor (VEGF) signalling pathway can result in hypertension. The frequency of adverse effects in patients receiving VEGFR inhibitors ranges from 11 to 45%. The prevalence of hypertension has been reported to vary between 17 and 42% in patients treated with sorafenib and between 15 and 47% in those treated with sunitinib. ^9, 10^ Anthracyclines, mitotic inhibitors, alkylating agents, proteasome inhibitors, and TKIs have all been linked to cardiotoxicity and symptoms, such as ischemia, arrhythmias, hypertension, LVD, and the symptoms of HF. ^11^ The European Society for Medical Oncology (ESMO) favors the use of angiotensin-converting enzyme (ACE) II inhibitors in asymptomatic patients with LVD who exhibit an ejection fraction of less than 40%. A proactive pharmacological approach is recommended for the management of hypertension associated with cancer treatment to reduce the risk of CV complications. In this particular situation, the most favored pharmacological agents for managing hypertension are ACE inhibitors and dihydropyridine calcium channel blockers. This assertion holds particularly true in cases where VEGFR therapy is utilized. ^15, 16^

The treatment of associated CVD in patients with NSCLC necessitates the use of multiple medications, leading to polypharmacy. Consequently, the potential for clinically significant drug-drug interactions (DDIs) is a crucial factor to be considered, particularly for patients who are prescribed multiple medications to manage comorbidities, as this may result in heightened toxicity or reduced therapeutic efficacy of the anticancer drugs. Empirical research conducted on cancer patients has recorded the interactions of TKIs with antiepileptic drugs, proton pump inhibitors, antiretroviral medications, antibiotics, and grapefruit juice, with an average rate of DDIs ranging from 4-40%. Furthermore, pharmacological interventions intended to manage cardiovascular toxicities that can also potentially result in DDIs. It is noteworthy that inquiries concerning such interactions are frequently omitted from preliminary clinical trials conducted during the initial phases of pharmaceutical development. Advancements in improving cancer survival rates are also sometimes overshadowed by pharmacokinetic/pharmacodynamic interactions associated with TKIs and medications used to treat concurrent CVD.

Therefore, the primary objective of this investigation was to identify plausible pharmacokinetic and pharmacodynamic DDIs in individuals diagnosed with NSCLC who are prescribed with TKIs and concomitant CV drugs through a comprehensive methodology that combines drug interaction checkers, IBM Micromedex ^®^, and Drugs.com ^®^, as well as an in-silico modelling technique for molecular docking. The integration of data obtained from the DDI checker software and in silico computational technique is anticipated to yield a more all-encompassing depiction and projection of probable DDIs linked to TKIs and CV medications. This will aid oncologists in adjusting dosages, selecting an optimal standard care protocol, and delivering optimal supportive care.

## 2. Methods

### 2.1 Identification of potential DDIs by IBM Micromedex software and Drugs.com

To identify potential DDIs between CV drugs and TKIs used in NSCLC treatment, all generic names were added to the drug interaction checkers, that is, IBM Micromedex ( https://www.micromedexsolutions.com/) and Drugs.com ( https://www.drugs.com/drug_interactions). Both interaction checking tools were used till July 30, 2024. The severity of potential pharmacokinetic and pharmacodynamic DDIs from IBM Micromedex was classified into five groups: contraindicated, major, moderate, minor, and no interaction reported. Drugs. com results were classified as major, moderate, or minor. The list of TKIs used in the treatment of NSCLC was compiled from the National Comprehensive Cancer Network (NCCN) Guidelines and Food and Drug Administration (FDA) approved NSCLC drugs. Whereas, the CV drugs prescribed for cancer patients with CVD were compiled from the ESMO ( https://www.esmo.org/guidelines) guidelines. ^16– 21^

### 2.2 Identification of potential DDIs by molecular docking

***2.2.1 Protein and ligand preparation***

The crystal structures of human pregnane X receptor (PXR) proteins (PDB ID: 5XOR) and the human ether-a-go-go-related gene (hERG) were retrieved from the Protein Data Bank (PDB) ( http://www.rcsb.org/pdb) and Schrödinger knowledgebase ( https://www.schrodinger.com/), respectively. The protein crystal structure was optimized using the Schrodinger 2021-2 Protein Preparation Wizard. Protein pre-processing was accomplished through bond order assignment, hydrogen addition, disulfide treatment, and the removal of water molecules from hetero groups larger than 5 Å. Hydrogen bonds were assigned, and the alignments of the remaining water molecules were adjusted. Finally, using the OPLS4 force field at pH 7.4, the energy of the protein structure was reduced to an RMSD of 0.30 Å. The co-crystallized ligand was retained in the prepared protein using the default parameters, which were then used for grid construction. As there was no bound ligand in the case of hERG, site map analysis was performed using the site map analysis module to identify the top-ranked receptor-binding sites of the enzyme. ^22^ In the case of PXR, since the protein was bound to an antagonist/activator, the same site was selected for molecular docking in the presence and absence of an antagonist. The structural details of the 22 TKIs and 76 CV drugs were retrieved from the PubChem database. Ligand preparation for TKIs and CV drugs was carried out at pH 7.4, using the Lig-prep application of the Maestro 11.08 module of the Schrodinger ^®^ suite 2021-2 in accordance with a previously reported procedure using the OPLS4 force field to minimize the energy of the structures and to correct the chirality. A grid was generated at the center of the PDB 5X0R co-crystallized ligand and top-ranked site of the hERG protein. Flexible molecular ligand docking was performed using extra precision (XP) mode and force field OPLS4. Ten poses per ligand were generated, and the RMSD values were calculated. The ligand position in the center of a 10 Å docking sphere was restricted. All molecules were also docked with the protein at the ligand-binding site using the Schrodinger suite-2021-2 Glide XP algorithm.

***2.2.2 Induced fit docking and PRIME MMGBSA analysis***

After XP docking, 16 TKIs and 45 CV drugs with the best docking scores and poses were selected for flexible induced-fit protein-ligand docking using the induced-fit docking (IFD) module of the Maestro 12.7 molecular modelling platform (generates up to 20 poses using an automatic docking protocol). The binding affinity of each TKI and CV drug to both PXR and hERG proteins was evaluated using the Prime-MMGBSA module of Schrödinger Suite 2021-2 (Schrödinger, LLC, New York, NY, 2021) for all poses generated by the IFD. The total free energy of binding, dGbind (Kcal/mol), was estimated using this software. A grid spacing of 0.5 Å was chosen, and the dielectric constants for the solute and solvent were set to 1 and 80, respectively. ^23^ The total energy term is a combination of Coulomb energy, covalent binding energy, van der Waals energy, lipophilic energy, prime energy, hydrogen-bonding energy, pi-pi packing energy, and self-contact correction. A scoring method was applied to calculate the net dG bind (NB), by calculating the mean IFD scores obtained for each ligand at different poses as described by Thomas et al. ^22^

## 3. Results

### 3.1 Identification of potential DDIs by Micromedex software and Drugs.com

The objective was to evaluate the potential DDIs of calcium channel blockers, ACE inhibitors, statins, cardiac glycosides, β-adrenergic blockers, and other drugs utilized in the management of CVD, in patients on TKI chemotherapy. Table 1 presents an extensive list of 75 significant pharmacokinetic and 102 crucial pharmacodynamic DDIs between TKIs and CV drugs. The table includes information on the severity of the interactions and the level of documentation obtained from IBM Micromedex and Drugs.com. Seven contraindicated pharmacokinetic and pharmacodynamic interactions were identified using CV drugs. Among the 76 CV drugs screened, 45 showed DDIs with TKIs, based on the severity of the interaction. Twenty-two DDIs were identified to have excellent documentation level, as per the IBM Micromedex data.

**Table 1.** Severity classification of pDDIs of CV drugs with TKIs.

Severity classification of pDDIs	Micromedex n (%)			Drugs.com n (%)		
	PK	PD	Both	PK	PD	Both
Contraindicated	N/A	6 (13.95)	N/A	N/A	N/A	N/A
Major	57 (91.9)	37 (86.04)	10 (100)	17 (7.76)	64 (57.1)	N/A
Moderate	5 (8.0)	N/A	N/A	202 (92.2)	48 (42.8)	N/A
Minor	N/A	N/A	N/A	N/A	N/A	N/A
Total number of pDDIs	62 (100)	43 (100)	10 (100)	219 (100)	112 (100)	N/A

* pDDIs: potential drug-drug interactions; N/A: not applicable; PK: Pharmacokinetic; PD: Pharmacodynamic.

***3.1.1 Pharmacokinetic interactions with PXR***

**Figure 1.**
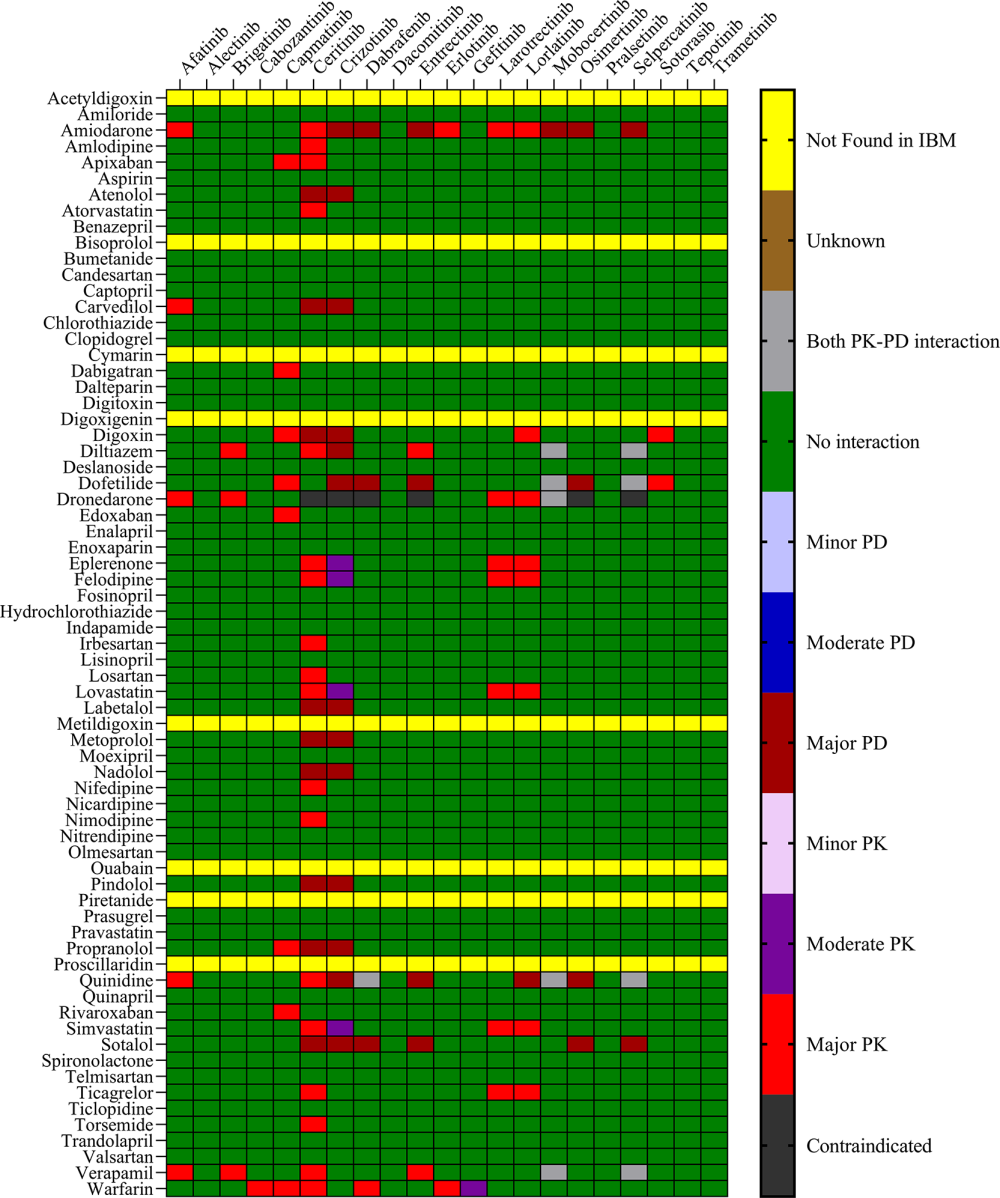
Severity chart for interactions between TKI and CV drugs identified by Micromedex ^®^ software.

**Figure 2.**
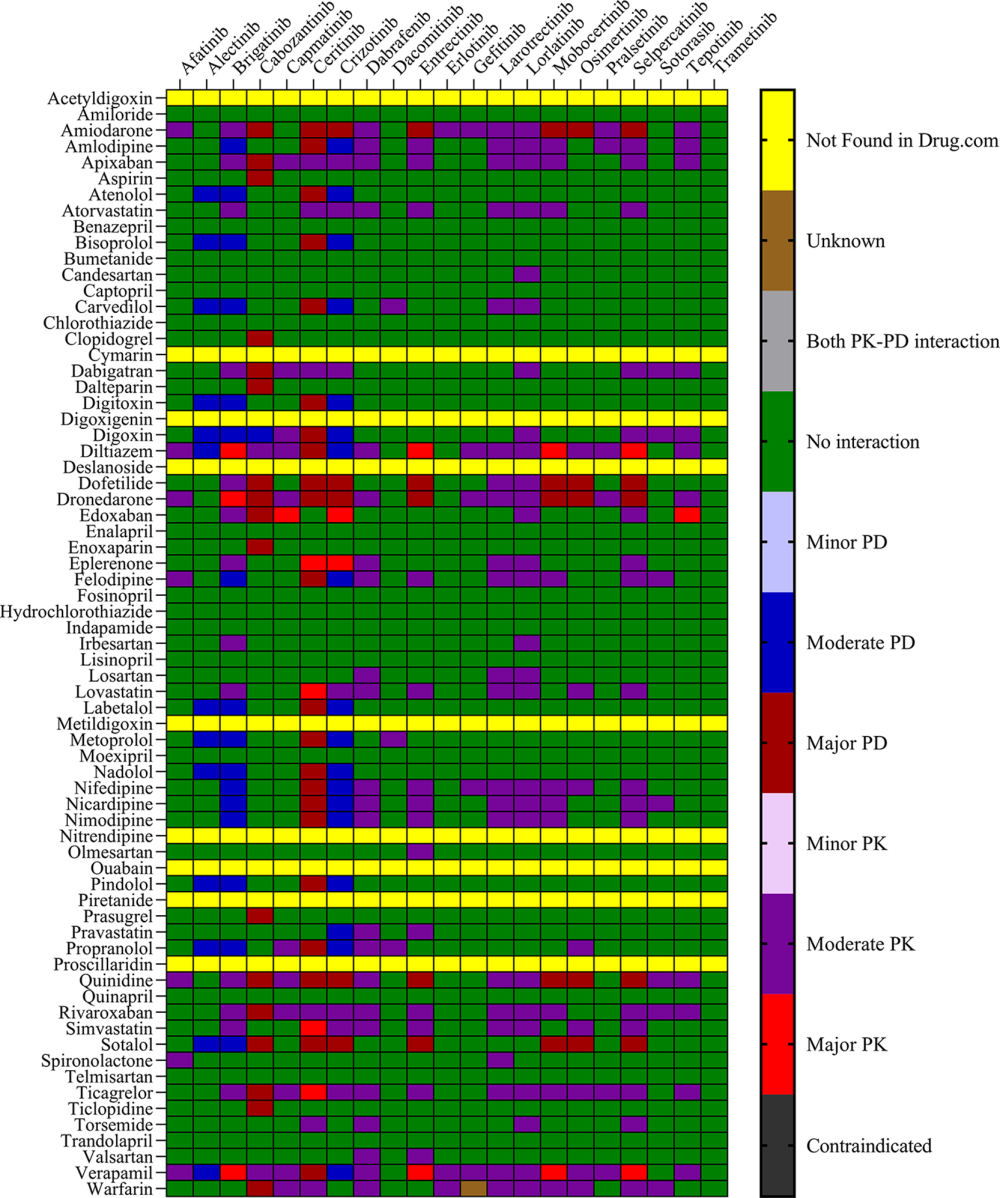
Severity chart for interactions between TKI and CV drugs identified by Drugs.com ^®^.

Sixteen TKIs and 45 CV drugs were found to exhibit significant pharmacokinetic DDIs based on analysis using IBM Micromedex software, Drugs.com, and the IFD docking score for both PXR and hERG proteins. The results are presented in Figures 1 and 2 for IBM Micromedex and Drugs. Com, respectively. Most of the observed pharmacokinetic interactions were attributed to the potential of CV drugs and TKIs to either inhibit or induce the PXR protein. Table 2 displays the net scores obtained from the IFD for TKIs and CV drugs. The molecular docking technique was used to determine the net binding score and establish the potency of these drugs in inhibiting PXR. Net binding scores were calculated for various TKIs using the induced fit docking of the PXR protein. The highest net binding scores were observed for tepotinib (NB = -880.57), cabozantinib (NB = -867.70), entrectinib (NB = -867.55), moboceritinib (NB = -855.66), osimeritinib (NB = − 844.43), trametinib (NB = -796.00), and brigatinib (NB = -785.75), as shown in Table 2). Previous studies (As represented in the Underlying dataset) have reported that these TKIs can function as substrates or inhibitors of both CYP450 and P-gp. ^24^

**Table 2.** Net binding energy of individual TKIs drugs and CV drugs with PXR and hERG proteins.

Drug Category	Compounds	PXR	hERG
**NSCLC TKIs**	Afatinib	-699.28	-440.25
	Alectinib	-744.33	-488.32
	Brigatinib	-785.75	-470.41
	Cabozantinib	-867.70	-543.82
	Capmatinib	-670.82	-460.27
	Ceritinib	-733.37	-484.40
	Crizotinib	-679.99	-348.19
	Dabrafenib	-511.28	-382.66
	Dacomitinib	-511.28	-382.66
	Entrectinib	-867.55	-523.75
	Erlotinib	-725.35	-459.49
	Gefitinib	-722.00	-448.05
	Larotrectinib	-647.03	-407.13
	Lorlatinib	-580.16	-322.69
	Mobocertinib	-855.66	-447.48
	Osimertinib	-844.43	-468.28
	Pralsetinib	-691.52	-425.55
	Selpercatinib	-699.42	-521.97
	Sotorasib	-733.97	-463.74
	Tepotinib	-880.57	-520.27
	Trametinib	-796.00	-494.30
**ACE inhibitors**	Fosinopril	-1798.77	-770.45
	Moexipril	-1266.99	-865.22
	Benazepril	-1445.26	-688.35
	Trandolapril	-1591.7725	-712.45
	Quinalapril	-1471.32	-746.18
	Lisinopril	-1263.38	-770.41
	Enalapril	-1201.64	NA
	Captopril	-591.09	-377.82
**Angiotensin II blockers**	Irbesartan	-1540.61	-655.82
	Losartan	-1584.54	-887.16
	Telmisartan	-2252.21	-1139.66
	Candesartan	-1010.3	-443.99
	Valsartan	-850.314	-416.80
	Olmesartan	-1521.57	-947.03
**Anticoagulant**	Apixaban	-2119.13	NA
	Edaxaban	-2176.09	-717.70
	Enoxaparin	NA	-52.02
	Rivaroxaban	-1901.58	-1248.11
**B adrenergic blockers**	Atenolol	-1485.54	-1115.21
	Bisoprolol	-1616.49	-1309.66
	Carvedilol	-2068.62	-1167.89
	Labetalol	-1195.43	-652.11
	Metoprolol	-1512.02	-1156.43
	Pindolol	-1303.94	-874.82
	Propranolol	-1408.91	-1125.55
	Sotalol	-1168.67	-999.75
**Ca Channel blocker**	Diltiazem	-1768.94	-1112.12
	Felodipine	-1398.12	-935.18
	Nifedipine	-1116.01	-924.97
	Nicardipine	-1878.41	-1244.34
	Nimodipine	-1289.5	-1087.17
	Verapamil	-2172.57	-1219.60
	Amlodipine	-1732.96	-1140.35
	Nitrendipine	-1475.34	-1010.26
**Dual antiplatelet agents**	Aspirin	-552.948	-1219.60
	Clopidogrel	-1550.55	NA
	Prasugrel	-1654.84	NA
	Warfarin	-1034.21	-579.85
**Statins**	Simvastatin	-1768.12	-877.75
	Pravastatin	-1351.41	-866.47
**Others**	Dabigatran	-1736.23	-1016.93
	Digoxin	NA	-771.36
	Dofetilide	-1691.74	-938.80
	Dronedarone	-2235.37	-1210.14
	Eplerenone	-1642.66	-869.22
	Quinidine	-1791.56	-1249.65
	Torsemide	-1149.07	-567.09
	Ouabain	-1766.71	-891.84
	Proscillaridin	-1764.54	-1094.61
	Amiodarone	-2081.18	-1144.70
	Cymarin	-1773.25	-941.75
	Digoxigenin	-1573.11	-964.18
	Indapamide	-1470.71	-986.82
	Hydrochlorothiazide	-880.518	-646.37
	Bumetanide	-979.594	-710.80
	Piretanide	-956.431	-546.96
	Spironolactone	-997.025	-451.19

* NA: Not enough poses to calculate binding energy.

The net binding energy was computed using the docking scores obtained from the IFD of distinct CV on the PXR protein. The compounds amiodarone, carvedilol, telmisartan, verapamil, dabigatran, amlodipine, nicardipine, fosinopril, olmesartan, edexaban, apixaban, and simvastatin exhibited favorable binding interactions with the PXR protein, as evidenced by their negative binding energies (-2081.18, -2068.62, -2252.21, -2172.57, -1736.23, -1732.96, -1878.41, -1798.77, -1521.57, -2176.09, -2119.13, and -1768.12 Kcal/mol respectively). Table 2 presents the net binding energies obtained through IFD of certain CV drugs. PXR has been characterized as a chemical sensor possessing a ligand-binding domain that exhibits flexibility, allowing for the accommodation and adaptation of various molecules. The PXR protein contains several key amino acid residues responsible for ligand binding. Specifically, TRP299, GLN285, MET323, HIS327, SER247, LEU209, MET243, PHE251, PHE281, CYS284, and MET323 are known to play crucial roles in this process. ^25, 26^ For CV drugs, the induced fit docking method was used to first calculate the docking score and then the binding energy. The results indicated that calcium channel blockers, specifically verapamil, nicardipine, diltiazem, amlodipine, and nifedipine, displayed the most substantial binding energies. Calcium channel blockers, which can affect the metabolism and absorption of TKIs, have been identified as potent inhibitors of CYP3A4 and P-gp. This can lead to pharmacokinetic DDIs when these two drugs are administered concurrently. In addition, various in vitro studies have demonstrated that antiarrhythmic and dual antiplatelet medications, including amiodarone, dronedarone, and ticagrelor, significantly inhibit CYP3A4 and P-gp. The binding energy obtained against the PXR protein supports the hypothesis made in the literature. ^27– 30^

**Figure 3.**
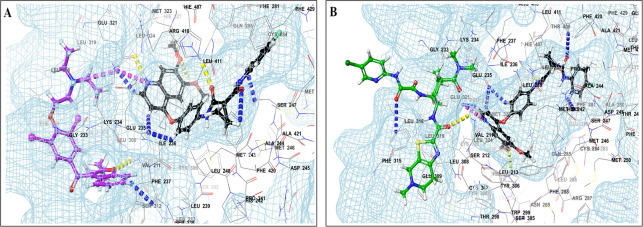
PXR protein showing PXR-LBD and AF-2 SRC-1 region. Binding mode of 3D interaction scheme at PXR LBD binding site near to AF-2 helix of cabozantinib co-docked pose with (A) amiodarone, and (B) edaxaban.

**Figure 4.**
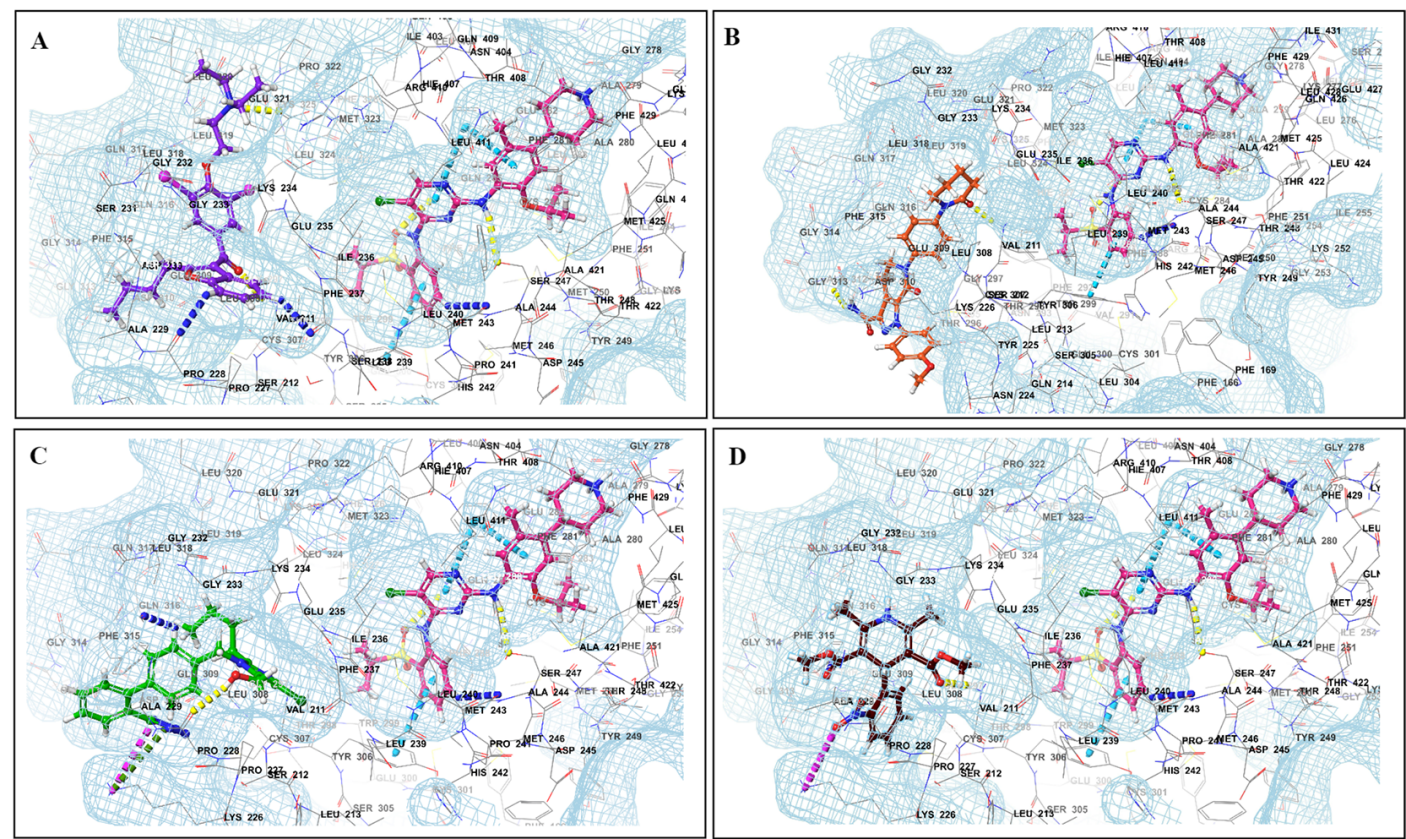
PXR protein showing PXR-LBD and AF-2 SRC-1 region. Binding mode of 3D interaction scheme at PXR LBD binding site near to AF-2 helix of ceritinib co-docked pose with (A) amiodarone, (B) apixaban, (C) losartan, and (D) nifedipine.

This study utilized a co-docking approach to investigate the capacity of CV drugs to interact with and activate PXR in the presence and absence of TKIs. The aim of this study was to observe any synergistic effects on the binding energies of the co-docked pose. The majority of interactions arise from the interaction between TKIs, whereas CV drugs either inhibit or induce CYP3A4 or P-gp. This can result in increased levels of TKIs in plasma. Co-docking analysis of PXR revealed an increase in the binding energy of specific CV drugs in the presence of TKIs at the docking site. Afatinib is both a substrate and inhibitor of P-gp. Co-docking studies have shown that the binding energies of amiodarone (BE = -48.01), spironolactone (BE = -23.13), proscillaridin (BE = -34.89), and telmisartan (BE = -25.48) increased synergistically when docked in combination with afatinib. Validation of this predicted result requires additional supportive clinical data. Cabozantinib is a reported substrate of CYP3A4 and an inhibitor of P-gp. The results of the co-docking studies revealed that the interactions with CV drugs, such as amiodarone, dabigatran, and edoxaban, were the most prominent ( Figure 3). This study revealed that the presence of ceritinib had a notable impact on the binding energies of several drugs, including amiodarone (BE = -43.84), nimodipine (BE = -39.63), apixaban (BE = -45.14), simvastatin (BE = -45.06), felodipine (BE = -40.29), torsemide (BE = -44.25), losartan (BE = -32.24), cymarin (BE = -42.25), lovastatin (BE = -44.7), telmisartan (BE = -35.25), warfarin (BE = -27.19), and proscillaridin (BE = -27.65). These interactions were significantly different from those observed with other TKIs and CV drugs ( Figure 4). Moboceritinib, a moderate inhibitor of both CYP3A4 and P-gp, exhibited significant pharmacokinetic interactions with amiodarone (BE = -50.15), proscillaridin (BE = -22.31), and telmisartan (BE = -27.62), all of which are known to be inhibitors of CYP3A4 and P-gp (As represented in the underlying dataset and Supplementary Tables S1 and S2).

***3.1.2 Pharmacodynamic interactions with hERG***

We identified 37 and 64 major pharmacodynamic interactions of TKIs with CV drugs in IBM Micromedex and Drugs.com, respectively ( Figures 1 and 2). Computational studies have shown that cabozantinib (NB = − 543.82), entrectinib (NB = -523.75), selpercatinib (NB = -521.97), ceritinib (NB = -484.40), osimeritinib (NB = -468.28), crizotinib (NB = -384.19), dabrafenib (NB = -382.66), gefitinib (NB = -448.05) and mobocertinib (NB = -447.48) have the maximum net binding scores to validate their binding affinity towards hERG. Although the net binding score of dabrafenib and crizotinib was not as high as that of the other TKIs, QT prolongation was reported in patients treated with these two TKIs (Represented in the Underlying dataset). The IFD results of individual CV drugs on hERG protein confirmed that telmisartan (NB = -1139.66), bisoprolol (NB = - 1309.66), nicardipine (NB = -1244.34), verapamil (NB = -1219.60), dabigatran (NB = -1016.93), dronedarone (NB = -1210.14), and amiodarone (NB = -1144.70) exhibited excellent binding interactions with hERG protein.

**Figure 5.**
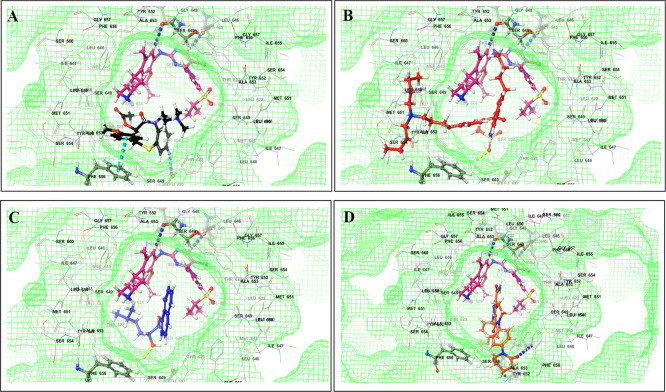
Binding mode of 3D interaction scheme at hERG binding site of ceritinib co-docked pose with (A) diltiazem, (B) dronedarone (C) propranolol and (D) apixaban.

**Figure 6.**
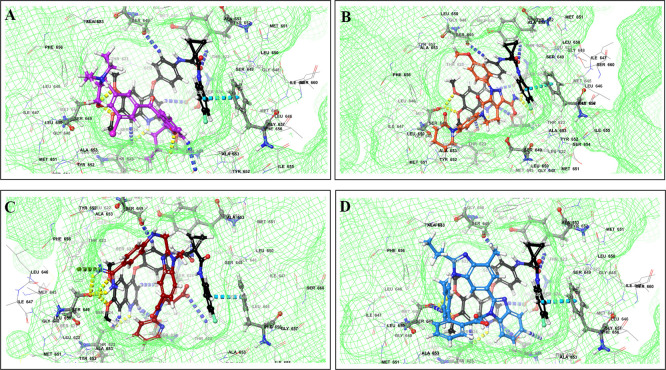
Binding mode of 3D interaction scheme at hERG binding site of cabozantinib co-docked pose with (A) amiodarone, (B) apixaban, (C) dabigatran and (D) ticagrelor.

The binding of different CV drugs, such as amlodipine (BE = -36.86), apixaban (BE = -44.57), diltiazem (BE = -53.69), bisoprolol (BE = -55.39), dofetilide (BE = -46.46), felodipine (BE = -45.84), labetalol (BE = -19.14), nifedipine (BE = -49.21), digoxigenin (BE = -42.69), verapamil (BE = -57.82), and oubain (BE = -40.54), was affected by the co-docking of hERG with TKIs, particularly ceritinib ( Figure 5). The co-docked pose of crizotinib with telmisartan (BE = -61.64), propranolol (BE = -44.42), and fosinopril (BE = -41.79) also demonstrated synergistic interactions ( Figure 6). According to the literature, crizotinib is also associated with the occurrence of bradycardia. TKIs, such as entrectinib and lorlatinib, also exhibited synergistic interactions with CV drugs, including amiodarone, dronedarone, digoxigenin, proscillaridin, trandolapril, and felodipine. Entrectinib and lorlatinib have also been documented to cause QT prolongation and PR interval prolongation in the therapy timeframe. The synergistic interactions between TKIs and CV drugs may increase the likelihood of cardiac arrest and other cardiac disease symptoms, as indicated in the Underlying dataset.

## 4. Discussion

According to the reports, a significant proportion of individuals diagnosed with lung cancer are of advanced age, with approximately 60% of patients falling within this demographic. Furthermore, up to 80% of this elderly cohort exhibited comorbidities, including but not limited to CVD, neuropsychiatric disorders, respiratory disorders, digestive disorders, and arthritis. CVD is commonly linked to chronic inflammation, which explains the observed correlation between these two medical conditions, and a report has also indicated a statistically significant association between the incidence of CVD and lung cancer. ^31, 32^ The observed trend can be primarily attributed to the significant and rapid increase in the number of therapies for orally administered anticancer agents, specifically molecular targeted treatments such as TKIs. According to the World Health Organization Drug Monitoring Centre’s collection of spontaneous adverse reaction reports, TKIs and 10 CV drugs were identified in 348 reports and were found to be linked to QT prolongation. ^33^ According to Waters et al. (2015), the prevalence of CYP3A-mediated interaction potential with TKIs and concurrently administered medications was 47%, 22%, and 11% for substrates, inhibitors, and inducers, respectively. ^34^ A retrospective cross-sectional analysis of adult patients with lung cancer in the United States Veterans Affairs healthcare system was conducted by Sawsan Rashdan et al. in 2021, and it was observed that 5.3% of patients who were administered concomitant medications had prescriptions for drugs linked to major DDIs, while 55.9% of patients had potential DDIs. ^35^

In a real-world study, FAERS database analysis focused on amivantamab, a dual-specificity EGFR/MET targeting agent, which revealed new insights into cardiovascular adverse events not captured in pre-approval clinical trials. Among nearly 20 million eligible reports, 98 cardiovascular events associated with amivantamab were identified, and disproportionality signal detection revealed statistically significant associations with venous thrombotic diseases, abnormal blood pressure, arrhythmias, and pericardial effusion. The median time-to-onset for these cardiovascular events was 33 days, with a high cumulative incidence of serious outcomes, including sudden death and major adverse cardiac events, within 90 days of therapy initiation. Additionally, another comprehensive pharmacovigilance analysis was carried out utilizing FAERS, which investigated cardiovascular toxicities linked to EGFR and VEGFR targeting TKIs, which are the most widely prescribed classes of targeted agents in oncology. This study analyzed over 12,000 cardiovascular adverse event reports and detected statistically significant safety signals across eleven TKIs, with hypertension emerging as the most commonly reported cardiovascular event. Notably, lenvatinib and sunitinib demonstrated strong associations with cardiac failure and cardiomyopathy, ponatinib with cardiomyopathy, and vandetanib with torsade de pointes/QT prolongation-risk patterns that varied by specific agent and event type. These real-world disproportionality findings underscore the heterogeneous cardiovascular risk profiles of VEGFR-TKIs and the importance of incorporating post-marketing pharmacovigilance data to more accurately characterize cardiotoxicity risks in clinical practice.

Thus, chemotherapy for NSCLC is a multidimensional regimen designed to address comorbidities and toxicity-related symptoms. This may lead to complications owing to the potential occurrence of pharmacokinetic and pharmacodynamic DDIs. Therefore, assessment of potential DDIs occurring with NSCLC medications and CV drugs can provide preliminary knowledge for screening, identification, and management of DDIs in these comorbid conditions. The study utilized drug interaction checkers and published literature to anticipate potential pharmacokinetic interactions between TKIs and concurrently administered drugs. No studies on pharmacokinetic DDIs of TKI with CV medications have been reported to assess the severity of the interaction with NSCLC therapy. Hence, in this study, we thoroughly evaluated the prevalence and risk of pharmacokinetic and pharmacodynamic interactions and their severity using computational tools and software such as IBM Micromedex and Schrödinger. Our study results revealed that the majority of pharmacokinetic/pharmacodynamic DDIs identified by IBM Micromedex were of major severity, whereas Drugs.com were of moderate severity. Synergistic inhibition/interference in the pathways of human PXR and hERG can give rise to both pharmacokinetic and pharmacodynamic interactions. Therefore, molecular docking studies assessed the docking scores and binding energy calculations in the binding pockets of both proteins for TKIs and CV drugs.

The study revealed that the interaction between hERG protein and various TKIs was prominent in second and third-generation TKIs, such as mobocertinib, dabrafenib, tepotinib, selpercatinib, brigatinib, cabozantinib, osimertinib, entrectinib, crizotinib, and afatinib. Ceritinib, crizotinib, and moboceritinib induce QT prolongation in patients. Apart from this, crizotinib was associated with bradycardic symptoms, whereas lorlatinib was found to prolong the PR interval. Additionally, gefitinib was directly linked to QT interval prolongation through hERG involvement, thereby supporting the findings of the molecular docking studies. The hERG channel also participates in several cellular processes, including apoptosis, angiogenesis, and cell proliferation. Wan et al. (2020) further investigated its role in cardiotoxicity and its potential as a therapeutic target in cancer physiology. ^36^ Moreover, Occhipinti M. et al. also reported that statin use was negatively correlated with progression-free survival in stage IV advanced NSCLC patients receiving EGFR-TKIs ( *p* = 0.02; HR 0.281, 95% CI: 0.096–0.825), indicating a detrimental drug interaction between statins and TKIs. ^37^

ACE inhibitors such as lisinopril and angiotensin II blockers, including telmisartan and olmesartan, as well as calcium channel blockers and β-blockers, are the primary CV drugs that interact with the hERG protein. These interactions are based on hydrophobic interactions and the net binding energy. In addition to the aforementioned drugs, dabigatran, dronedarone, and quinidine exhibited a significant affinity for the hERG protein. In 2005, Farid et al. conducted a study on the homo-tetrameric pore domain of hERG. This study aimed to investigate potential energy mapping and binding possibilities. The results showed that compounds containing hydrophobic groups, such as terfenadine, cisapride, sertindole, and ibutilide, exhibited strong binding properties. These findings suggested that the tetrameric ring may act as a strong binding pore. ^38^ Zhang et al *.* (2016) established a study validating the interaction between amiodarone and hERG potassium channel blocker pore with mutagenesis and its in vitro IC _50_ for the F656A mutation. ^39^ Additionally, Munawar et al. (2019) validated the involvement of potassium ions and water molecules in the hERG channel in the binding of the drug to the pore, which might be the possible reason for the difference in selectivity between drugs belonging to the same class. ^40^ Co-docking studies of TKIs revealed that ceritinib and cabozantinib were particularly susceptible to interactions with CV drugs, including diltiazem, dronedarone, propranolol, apixaban, amiodarone, dabigatran, and ticagrelor. Concurrent use of TKIs in combination with calcium channel blockers, dual antiplatelet agents, and ACE II inhibitors may result in cardiac arrhythmia and increase the likelihood of QT interval prolongation. Studies have reported that certain drugs, including amiodarone, quinidine, sotalol, and dofetilide, are also associated with an increased risk of QT interval prolongation.

Several TKIs and CV drugs have been reported to increase QTc interval in patients. ^33, 41, 42^ There is an increased risk of developing DDIs among cancer patients receiving TKI drugs with the concomitant use of CV drugs. ^43, 44^ Most pharmacodynamic DDIs identified in our study from IBM Micromedex were of major severity, whereas pharmacodynamic DDIs identified from Drugs.com had a significant proportion of both major and moderate severity. It was evident from the results obtained from IBM Micromedex and drug.com interactions checker that CV drugs such as amiodarone, dronedarone, quinidine, propranolol, and sotalol have shown potential for pharmacodynamic interactions ( Figures 1 and 2). The majority of drugs cause QTc interval prolongation by inhibition of the hERG subunit of the channel that conducts major ventricular repolarizing potassium current (IKr) during phases 2–3 of the action potential. ^45, 46^ In TKI-initiated cancer patients with concomitant CV drug intake, regular ECG monitoring is strongly recommended. Prospective studies evaluating QTc prolongation among cancer patients on the concomitant use of TKIs and CV drugs are needed to establish conclusive evidence.

In 2014, Van Leeuwen et al. emphasized that to ensure the safe administration of TKIs in clinical oncology, it is essential to evaluate the co-prescribed medications, herbal supplements, and dietary components such as grapefruit juice, as well as cardiac risk factors and findings from physical examinations. ^47^ Several clinical studies, such as those conducted by Akbulut et al. (2022) and Amina Haouala et al. (2010), have also reported DDIs between CV drugs and anticancer agents, including traditional chemotherapies as well as oral chemotherapies such as TKIs (crizotinib, afatinib, ceritinib). ^43, 48^ Due to their prolonged use and classification as substrates of CYP450 enzymes and efflux transporters such as P-gp and BCRP, patients treated with TKIs are at an increased risk of pharmacokinetic DDIs. Moreover, TKIs employed in the management of NSCLC, such as ceritinib, afatinib, cabozantinib, and crizotinib, have also been identified as inhibitors of CYP3A4 and P-gp, elevating the risk of pharmacokinetic DDIs. ^49^

The results of the DDI checkers and molecular docking analyses indicated that these drugs have a high binding affinity to the ligand-binding domain of the PXR protein, evidenced by their high binding energies, as shown in Figure 2 and Table 2. Similarly, it was evident from the results of the drug interaction checker softwares that CV drugs such as calcium channel blockers, β-blockers, anticoagulants, and angiotensin II blockers have the potential to cause major pharmacokinetic interactions and the potential to inhibit the PXR protein. The net binding energies of these inhibitors ranged from -1500 to -2400 Kcal/mol. Calcium channel blockers and β-blockers are known for their ability to inhibit CYP3A4. ^50, 51^ Concurrent use of these drugs may substantially increase the plasma concentration of TKIs. In a crossover study involving healthy volunteers, Teng et al. (2013) investigated the pharmacokinetic interactions between diltiazem and CYP3A4 substrates. The results of this study indicate that diltiazem significantly inhibits CYP3A4, resulting in pharmacokinetic DDIs. ^52^ Hukkanen et al. (2015) also reported the inhibitory activity of atorvastatin on PXR protein, indicating the potential of statins as drugs for the occurrence of DDIs. ^53^

Information derived from both IBM Micromedex and Drugs.com revealed that several pharmacokinetic DDIs between statins and TKIs of major and moderate severity were also identified in both databases. The results of the binding energy calculations were also in the same line, where simvastatin (BE = -33.39), lovastatin (BE = -33.36), and pravastatin (BE = -41.66) showed prominent interactions with dabrafenib, ceritinib, crizotinib, larotrectinib, and lorlatinib. DDIs between these statins and TKIs were detected using both drug information databases. The drug information databases have also identified several other antihypertensive drugs, including telmisartan, nifedipine, carvedilol, amlodipine, dabigatran, amiodarone, and felodipine, as drugs having major and moderate severity DDIs with TKIs. Computational assessments indicated that the drugs exhibited robust interactions with PXR both independently and when combined, as revealed through docking results. The computational predictions presented in this study offer clinically actionable insights for managing NSCLC patients with cardiovascular comorbidities. Identification of the high-risk DDI pairs between TKIs and commonly prescribed CV agents will aid clinicians in making more informed decisions regarding therapeutic combinations. These findings align with current NCCN and ESMO recommendations and may support the development of personalized treatment regimens that integrate pharmacokinetic/pharmacodynamic risk profiles into routine oncology practice. Further research is necessary to determine the clinical significance of these DDIs. This research will help to establish conclusive evidence and develop guidelines for the identification, monitoring, and management of DDIs in clinics.

## 5. Conclusion

The current study reveals that, the concurrent use of TKIs and CV drugs in the treatment of NSCLC presents a risk for clinically significant DDIs. These interactions were predicted using established clinical DDI checkers and validated using the in silico molecular docking technique. The predicted DDIs, especially those involving CYP3A4 modulation, P-gp transport, and QT prolongation, highlight clinically relevant risks that may compromise treatment safety. The findings underscore the importance of a comprehensive treatment strategy, particularly for NSCLC patients with coexisting cardiovascular conditions or chemotherapy-induced cardiac toxicity. Collaborative efforts among oncologists, cardiologists, and pharmacists are critical for identifying and managing this category of DDIs in cancer patients. These insights support the need for multidisciplinary management and warrant further prospective studies in a clinical setting to design a precise and informed prescribing practice for optimisation of therapeutic outcomes. The data resulting from this study can inform the development of a robust surveillance framework for monitoring DDIs during TKI therapy, ultimately improving clinical outcomes, reducing healthcare costs, minimizing morbidity, and enhancing patients’ quality of life.

## Author contributions

**Prajakta Harish Patil:** Conceptualization of the study, collection of data, and writing of the manuscript. **Mrunal Pradeep Desai:** Collection and interpretation of data. **Gayathri Baburaj:** Collection and interpretation of data. **Levin Thomas:** Collection and interpretation of data. **Viswam Subeesh:** Collection and interpretation of data and review of the manuscript. **Sumit Birangal:** Collection and interpretation of data. **Mahadev Rao:** Data review and manuscript review. **Gurupur Gautham Shenoy:** Interpretation of data, review of data, and review of manuscript. **Jagadish P. C.:** Conceptualization of study, Review of data, Review of manuscript.

## Data Availability

Figshare: Optimizing Cardiovascular Treatment in Non-Small Cell Lung Cancer: A Comprehensive Computational Approach for Assessment of Drug-Drug Interactions between Tyrosine Kinase Inhibitors and Cardiovascular Drugs, DOI: https://doi.org/10.6084/m9.figshare.28451273. ^54^ This project contains the following underlying data:
• IBM Micromedex and Drugs.com with available reported literature: https://doi.org/10.6084/m9.figshare.28451273
○
Figure 1 ○
Figure 2 ○ Raw data_1_NSCLC_DDI_Literature
The above-mentioned dataset represents the literature related data and also the data retrieved from IBM Micromedex and drugs.com
Data are available under the terms of the Creative Commons Attribution 4.0 International license (CC-BY 4.0). Figshare: Optimizing Cardiovascular Treatment in Non-Small Cell Lung Cancer: A Comprehensive Computational Approach for Assessment of Drug-Drug Interactions between Tyrosine Kinase Inhibitors and Cardiovascular Drugs, DOI: https://doi.org/10.6084/m9.figshare.28512680.v1. ^55^ The project contains the following extended data:
• F1000_Supplementary file 1_JPC Data are available under the terms of the Creative Commons Attribution 4.0 International license (CC-BY 4.0).

## Software availability statement

In this study, IBM Micromedex and Schrödinger softwares were utilized for data analysis and molecular docking, respectively. IBM Micromedex is a paid subscription-based software accessible through the university. However, similar data can be obtained from Drugs.com, which is a freely available resource. Schrödinger was used for molecular docking and modelling. This is also a paid software. However, an open-source alternative, AutoDock, can be used for molecular modelling.
