# Supplementary material for: Optimizing Cardiovascular Treatment in Non-Small Cell Lung Cancer: A Comprehensive Computational Approach for Assessment of Drug-Drug Interactions Between Tyrosine Kinase Inhibitors and Cardiovascular Drugs
Source: F1000Res. 2026 Feb 2;14:309. Originally published 2025 Mar 19. [Version 3] doi: 10.12688/f1000research.162353.3 (PMC12744204; doi:10.12688/f1000research.162353.3)
Supplement: Peer review history

# Reviewer report: Reviewer response for version 2

**DOI:** 10.5256/f1000research.189728.r431630

**Published:** 2025-12-27

**Authors:** Janmejay Pant

**Affiliations:** 1 Chandigarh University, Sahibzada Ajit Singh Nagar, Punjab, India

The manuscript is well-written and demonstrates good scientific value. Overall, it can be considered for acceptance after the authors address the following comments. In particular, it is important to incorporate the most recent pharmacoepidemiology data to ensure that the findings remain current, relevant, and aligned with recent developments in the field. Updating the data sources will significantly strengthen the validity and overall impact of the manuscript.

Is the work clearly and accurately presented and does it cite the current literature?

Yes

If applicable, is the statistical analysis and its interpretation appropriate?

Not applicable

Are all the source data underlying the results available to ensure full reproducibility?

Yes

Is the study design appropriate and is the work technically sound?

Yes

Are the conclusions drawn adequately supported by the results?

Yes

Are sufficient details of methods and analysis provided to allow replication by others?

Yes

Reviewer Expertise:

Non-Small lung cancer cell line

I confirm that I have read this submission and believe that I have an appropriate level of expertise to confirm that it is of an acceptable scientific standard.

# Reviewer report: Reviewer response for version 2

**DOI:** 10.5256/f1000research.189728.r426077

**Published:** 2025-10-31

**Authors:** Fahrul Nurkolis

**Affiliations:** 1 Pharmaceutical, Nutritional, Biomedical, and Translational Medicine, Medical Research Center of Indonesia, Surabaya, East Java, Indonesia; 2 State Islamic University of Sunan Kalijaga, Yogyakarta, Yogyakarta, Indonesia; 3 Master of Basic Medical Science, Faculty of Medicine, Airlangga University (Ringgold ID: 148005), Surabaya, East Java, Indonesia

Thank you for your detailed and thoughtful responses. While I appreciate the clarification regarding computational limitations, I would still strongly recommend performing **at least one Molecular Dynamics (MD) simulation** for validation purposes. Even a single representative complex—such as one promising TKI–CV drug pair (e.g., *ceritinib–nicardipine*, *cabozantinib–verapamil*, or *brigatinib–amiodarone*)—across the **three key target proteins (PXR, hERG, and one additional receptor of clinical interest)** would substantially strengthen the manuscript.

 Including such MD data, even as a focused case study, would:

1. Demonstrate the **stability and dynamic interaction behavior** of the predicted complexes.
2. Provide **proof-of-concept evidence** supporting the broader docking results.
3. Increase the manuscript’s **scientific rigor and translational value**.

Therefore, I strongly encourage the authors to include **one concise MD simulation** to reinforce the robustness of their computational findings.

Is the work clearly and accurately presented and does it cite the current literature?

Partly

If applicable, is the statistical analysis and its interpretation appropriate?

Partly

Are all the source data underlying the results available to ensure full reproducibility?

Yes

Is the study design appropriate and is the work technically sound?

No

Are the conclusions drawn adequately supported by the results?

Partly

Are sufficient details of methods and analysis provided to allow replication by others?

Partly

Reviewer Expertise:

Molecular Biology, Pharmacoinformatics, In Silico, Drugs Discovery, Natural Product, Metabolic Syndrome and Anticancer

I confirm that I have read this submission and believe that I have an appropriate level of expertise to confirm that it is of an acceptable scientific standard, however I have significant reservations, as outlined above.

# Reviewer report: Reviewer response for version 1

**DOI:** 10.5256/f1000research.178544.r388784

**Published:** 2025-10-07

**Authors:** Uday Venkat Mateti

**Affiliations:** 1 NGSM Institute of Pharmaceutical Sciences (NGSMIPS), Nitte (Deemed to be University), Mangalore, Karnataka, India

1. What is the rationale for choosing clinical DDI checkers (Micromedex & Drugs.com)

 2. What is the rationale for choosing only PXR and hERG proteins?

 3. The method outlines MMGBSA binding energy calculations but does not define thresholds or benchmarks for classifying strong versus weak interactions, making interpretation potentially ambiguous.

 4. The conclusion should be made more concise by directly linking the key findings of predicted DDIs with their clinical implications, while briefly acknowledging the need for clinical validation and multidisciplinary management.

Is the work clearly and accurately presented and does it cite the current literature?

Yes

If applicable, is the statistical analysis and its interpretation appropriate?

Yes

Are all the source data underlying the results available to ensure full reproducibility?

Yes

Is the study design appropriate and is the work technically sound?

Yes

Are the conclusions drawn adequately supported by the results?

Yes

Are sufficient details of methods and analysis provided to allow replication by others?

Yes

Reviewer Expertise:

Patient Reported Outcomes and Cancer Research

I confirm that I have read this submission and believe that I have an appropriate level of expertise to confirm that it is of an acceptable scientific standard.

# Response

**Published:** 2025-10-14

**Authors:** Jagadish P. C.

**Affiliations:** Pharmaceutical Chemistry, Manipal Academy of Higher Education, Manipal, Karnataka, India

**Dear Reviewer,**

 Thank you for your insightful suggestions regarding our published manuscript entitled *“Optimizing Cardiovascular Treatment in Non-Small Cell Lung Cancer: A Comprehensive Computational Approach for Assessment of Drug-Drug Interactions between Tyrosine Kinase Inhibitors and Cardiovascular Drugs”* in F1000 Research. We have carefully revised the manuscript in accordance with your recommendations and corrections. We are confident that these improvements enhance the overall quality of the research. Respective changes have been modified and included in the revised version of the manuscript:

**Response to comments**

**1. **What is the rationale for choosing clinical DDI checkers (Micromedex & Drugs.com)

**Author Response: **Dear Reviewer, thank you for your comment regarding the rationale behind selecting Micromedex and Drugs.com as DDI checkers. IBM Micromedex is a peer-reviewed, evidence-based DDI checker widely used in hospital settings for generating official drug interaction risk reports, which is also a subscription-based platform. In contrast, Drugs.com is a freely accessible online interface that reports interactions based on literature and possible common real-world screening practices among clinicians. By integrating both platforms, we aimed to confirm clinically relevant drug–drug interactions through evidence-based sources as well as those frequently encountered in routine practices. This approach also enabled us to contextualize our molecular docking findings against known clinical interactions and explore mechanistic insights for unreported DDIs. The details of the same are outlined in the methodology section of the manuscript. (Page no. : 06-08)

**2. **What is the rationale for choosing only PXR and hERG proteins?

**Author Response: **Dear Reviewer, the selection of the Pregnane X Receptor (PXR) and the human Ether-à-go-go-Related Gene (hERG) channel as molecular docking targets was driven by their central roles in the two most clinically significant classes of DDIs in cardio-oncology, i.e., pharmacokinetic (PK) and pharmacodynamic (PD) interaction risks, respectively. While PXR governs key metabolic pathways influencing PK interactions, hERG is critical for assessing PD-related cardiotoxicity. This focused approach ensured that our computational predictions remain directly aligned with clinically actionable risk assessment in NSCLC patients with cardiovascular comorbidities. The details of the same are outlined in the methodology section of the manuscript. (Page no. : 06-08)

**3. **The method outlines MMGBSA binding energy calculations but does not define thresholds or benchmarks for classifying strong versus weak interactions, making interpretation potentially ambiguous.

**Author Response: **Dear Reviewer, in the current study, the MMGBSA binding energy, reported as Net Binding Energy, serves as an indicator of the thermodynamic favorability of drug-target interactions. Where a more negative (i.e., lower) MMGBSA value reflects a stronger and more favorable interaction, which in turn suggests a higher potential for DDI risk. This principle forms the basis for interpreting all subsequent binding data presented in the manuscript.

**4. **The conclusion should be made more concise by directly linking the key findings of predicted DDIs with their clinical implications, while briefly acknowledging the need for clinical validation and multidisciplinary management.

**Author Response: **Dear reviewer, thank you for the insightful suggestion. We have revised the conclusion section, acknowledging the need for clinical validation with a multidisciplinary patient care approach. (Page no: 17)

# Reviewer report: Reviewer response for version 1

**DOI:** 10.5256/f1000research.178544.r374190

**Published:** 2025-04-26

**Authors:** Fahrul Nurkolis

**Affiliations:** 1 Pharmaceutical, Nutritional, Biomedical, and Translational Medicine, Medical Research Center of Indonesia, Surabaya, East Java, Indonesia; 2 State Islamic University of Sunan Kalijaga, Yogyakarta, Yogyakarta, Indonesia; 3 Master of Basic Medical Science, Faculty of Medicine, Airlangga University (Ringgold ID: 148005), Surabaya, East Java, Indonesia

I have reviewed the manuscript entitled "Optimizing Cardiovascular Treatment in Non-Small Cell Lung Cancer: A Comprehensive Computational Approach for Assessment of Drug-Drug Interactions between Tyrosine Kinase Inhibitors and Cardiovascular Drugs" by Patil et al. with great interest. However, this paper requires extensive revision before it can meet the standards for indexing by reputable scientific databases. Below are detailed suggestions for improving the manuscript:

1. **Inclusion of Molecular Dynamics Simulations**: While molecular docking provides preliminary insights into interactions, the paper's current data should be complemented with Molecular Dynamics (MD) simulations. MD simulations would offer dynamic and temporal data on the stability of interactions identified by docking, providing deeper mechanistic insights and strengthening the manuscript's scientific rigor.
2. **Clarification of Drug Selection Rationale: **The authors need to provide a clear rationale for selecting specific cardiovascular drugs and tyrosine kinase inhibitors (TKIs). It is currently unclear why these particular drugs were chosen and why combinations with potentially more effective natural compounds or phytochemicals were not explored. Given the increasing interest and potential of natural compounds in therapeutic synergy, this oversight should be addressed explicitly.
3. **Extensive Proofreading for Grammar and Typographical Errors: **The manuscript contains several typographical and grammatical errors, which detract from readability and professionalism. For example, the term "GLOBACON" is incorrectly used in place of "GLOBOCAN". It is strongly recommended that proofreading be done by a professional proofreading service. Correcting these issues is crucial for enhancing the manuscript's clarity and impact.
4. **Expansion of Clinical Relevance**: While the computational data provided are robust, the manuscript would significantly benefit from discussions linking these computational predictions more explicitly to clinical contexts. A clearer interpretation of how these findings can practically influence treatment strategies or guidelines for clinicians managing NSCLC patients with cardiovascular comorbidities should be included.
5. **Enhancement of Figures and Tables Clarity**: The manuscript would benefit from clearer and more informative visual presentations. Figures and tables must be revised to ensure that they can independently convey key results. Improved annotations, legends, and explanations for complex data in the visual elements will substantially enhance comprehensibility.

In summary, addressing these major points will greatly enhance the manuscript’s scientific depth and applicability, thereby improving its potential for indexing in reputable scientific databases.

Is the work clearly and accurately presented and does it cite the current literature?

Partly

If applicable, is the statistical analysis and its interpretation appropriate?

Partly

Are all the source data underlying the results available to ensure full reproducibility?

Yes

Is the study design appropriate and is the work technically sound?

No

Are the conclusions drawn adequately supported by the results?

Partly

Are sufficient details of methods and analysis provided to allow replication by others?

Partly

Reviewer Expertise:

Molecular Biology, Pharmacoinformatics, In Silico, Drugs Discovery, Natural Product, Metabolic Syndrome and Anticancer

I confirm that I have read this submission and believe that I have an appropriate level of expertise to confirm that it is of an acceptable scientific standard, however I have significant reservations, as outlined above.

# Response

**Published:** 2025-10-14

**Authors:** Jagadish P. C.

**Affiliations:** Pharmaceutical Chemistry, Manipal Academy of Higher Education, Manipal, Karnataka, India

**Dear Reviewer,**

 Thank you for your insightful suggestions regarding our published manuscript entitled *“Optimizing Cardiovascular Treatment in Non-Small Cell Lung Cancer: A Comprehensive Computational Approach for Assessment of Drug-Drug Interactions between Tyrosine Kinase Inhibitors and Cardiovascular Drugs”* to F1000 Research. We have carefully revised the manuscript in accordance with your recommendations and corrections. We are confident that these improvements enhance the overall quality of the research. Respective changes have been modified and included in the manuscript:

**Response to comments**

**1. **Inclusion of Molecular Dynamics Simulations: While molecular docking provides preliminary insights into interactions, the paper's current data should be complemented with Molecular Dynamics (MD) simulations. MD simulations would offer dynamic and temporal data on the stability of interactions identified by docking, providing deeper mechanistic insights and strengthening the manuscript's scientific rigor.

**Author Response: **Dear Reviewer, thank you for your thoughtful suggestion. We agree that integrating MD simulations would significantly enhance the understanding of the study by providing dynamic and temporal insights into drug–protein interactions. However, due to the extensive computational resources and time required for a full MD study across the enormous number of drug combinations analyzed (22 TKIs and 76 CV drugs on both PXR and hERG), we view this essential investigation as a critical future scope of study. In future work, the study will be further continued to narrow down potential drug combinations (ceritinib–nicardipine, cabozantinib–verapamil, and brigatinib–amiodarone), which will also be shortlisted for subsequent **in vitro** and MD studies.

**2. **Clarification of Drug Selection Rationale: The authors need to provide a clear rationale for selecting specific cardiovascular drugs and tyrosine kinase inhibitors (TKIs). It is currently unclear why these particular drugs were chosen and why combinations with potentially more effective natural compounds or phytochemicals were not explored. Given the increasing interest and potential of natural compounds in therapeutic synergy, this oversight should be addressed explicitly.

**Author Response: **Dear reviewer, thank you for the valuable suggestion. The selection of drugs in our study was guided by clinical relevance, with the aim of providing immediate insights. We focused exclusively on agents that are currently being used as standard of care, i.e., tyrosine kinase inhibitors (TKIs), which were chosen based on NCCN and FDA-approved treatments for NSCLC, while cardiovascular drugs were selected according to ESMO guidelines for managing cardiovascular comorbidities in oncology settings, as detailed in the methodology section (Davis, 2025). At present, natural compounds fall beyond the scope of this study, but we recognize their potential and will consider them as a promising direction for future research. (Page no. 06)

**3. **Extensive Proofreading for Grammar and Typographical Errors: The manuscript contains several typographical and grammatical errors, which detract from readability and professionalism. For example, the term "GLOBACON" is incorrectly used in place of "GLOBOCAN". It is strongly recommended that proofreading be done by a professional proofreading service. Correcting these issues is crucial for enhancing the manuscript's clarity and impact.

**Author Response: **Respected reviewer, thank you for highlighting the typographical and grammatical issues in the manuscript. We sincerely apologize for these oversights. The manuscript has now undergone thorough proofreading to correct such errors and improve overall clarity.

**4. **Expansion of Clinical Relevance: While the computational data provided are robust, the manuscript would significantly benefit from discussions linking these computational predictions more explicitly to clinical contexts. A clearer interpretation of how these findings can practically influence treatment strategies or guidelines for clinicians managing NSCLC patients with cardiovascular comorbidities should be included.

**Author Response: **Dear reviewer, thank you for emphasizing the importance of clinical relevance. We agree that bridging computational predictions with practical clinical applications is essential for translational impact. Accordingly, we have modified the discussion section with relevant citations to correlate how our findings may inform treatment strategies for NSCLC patients with cardiovascular comorbidities. (Page no: 13-15, 17)

**5.**Enhancement of Figures and Tables Clarity: The manuscript would benefit from clearer and more informative visual presentations. Figures and tables must be revised to ensure that they can independently convey key results. Improved annotations, legends, and explanations for complex data in the visual elements will substantially enhance comprehensibility.

**Author Response: **Dear Reviewer, thank you for the suggestion. We have revised all figures to improve clarity, and high-resolution versions have been uploaded with the revised manuscript.
